# Blastocyst cryopreservation and cryopreservation-warming transfer is an effective embryo transfer strategy for day 1 rescue intracytoplasmic sperm injection cycles

**DOI:** 10.1038/s41598-021-87693-y

**Published:** 2021-04-15

**Authors:** Ming Li, Qin Li, Ying Wang, Jin Huang, Ping Liu

**Affiliations:** 1 Center for Reproductive Medical, Department of Obstetrics and Gynecology, Peking University Third Hospital, No. 49 North Huayuan Road, Haidian District, Beijing, 10091 China; 2 Key Laboratory of Assisted Reproduction (Peking University), Ministry of Education, Beijing, 10091 China; 3 Beijing Key Laboratory of Reproductive Endocrinology and Assisted Reproduction Technology, Beijing, 100191 China; 4 National Clinical Research Center for Obstetrics and Gynecology, Beijing, 10091 China

**Keywords:** Reproductive disorders, Microbiology techniques

## Abstract

This was a retrospective analysis of a total of 625 r-ICSI cycles using freeze-all-embryos and embryo transfers (ET) in subsequent cryopreservation-warming cycles to determine the effect of the ET method for day 1 rescue intracytoplasmic sperm injection cycles (r-ICSI). Two methods were used: in method 1, cleavage-stage embryos were frozen and were directly transferred in a subsequent cryopreservation-warming cycle (r-ICSI frozen cleavage), and 144 cleavage-stage ETs occurred. Similarly, in method 2, there were 188 blastocyst-stage ETs (r-ICSI frozen blast) performed. The live birth rate (LBR) for r-ICSI frozen blast was better than that for r-ICSI frozen cleavage in calculation of ET cycles (19.44% vs. 37.77%) and also remained better after the use of logistic regression analysis (OR = 2.721, 95% CI 1.604–4.616). Conservative cumulative LBR were compared between r-ICSI frozen cleavage and r-ICSI frozen blast with regard to oocyte retrieval cycles (17.39% vs. 15.30%). The same results were obtained for conservative cumulative LBR after logistic regression analysis (OR = 0.925, 95% CI 0.557–1.535). The results of this study confirmed that it was valuable to perform r-ICSI if using freeze-all-embryos. Further, r-ICSI embryos were cultured to blastocyst stage, cryopreserved, and used in subsequent cryopreservation-warming cycles, which was an effective embryo transfer strategy and obtained satisfactory results.

## Introduction

Total fertilization failure (TFF) often occurs unavoidably in conventional IVF cycles, even though human-assisted reproduction technology has been developed over decades. In order to salvage fertilization failure cycles, intracytoplasmic sperm injection (ICSI) of unfertilized mature oocytes (rescue ICSI (r-ICSI)) has been frequently recommended^1–8^. At present, there are two main methods of r-ICSI: D0 r-ICSI (early r-ICSI) and D1 r-ICSI (late r-ICSI). Early r-ICSI is performed on D0 oocyte retrieval and late r-ICSI is performed on D1 oocyte retrieval. Many studies have shown that r-ICSI on unfertilized 1-day-old mature oocytes (late r-ICSI) has been improved by performing earlier r-ICSI at 4–6 h (early r-ICSI) after insemination to optimize patient benefit, and early r-ICSI is a priority choice in clinics^5–8^. However, because early r-ICSI is performed beyond the daily working hours, many IVF units still adopt late r-ICSI. The current research focuses on late r-ICSI, and in the following r-ICSI only represents late r-ICSI.

Many embryologists believe that that r-ICSI has no clinical application because some studies have confirmed that the outcome of r-ICSI was poor on account of low clinical pregnancy rates, but it is worth noting that those studies were based only on cleavage embryo transfer (ET) in the fresh cycle^1,2^. Two main causes contribute to this finding: the low development potential of r-ICSI embryos, which is due to negative effects from the in vitro aging of cultured oocytes before ICSI, and the asynchrony between the developmental stage of the embryo and the endometrial secretory pattern when the embryo is transferred.

Some studies have evaluated the asynchrony between an embryo’s developmental stage and the endometrial secretory pattern, and it was found that r-ICSI embryos that are cryopreserved and subsequently used in cryopreservation-warming cycles can result in an acceptable pregnancy rate (PR)^9,10^. However, as reported in a previous study^9^, the approximately 25–30% PR achieved in cryopreservation-warming cycles using r-ICSI is not a satisfactory clinical outcome. This may be due to the transfer of cleavage embryos, which cannot guarantee good development potential, and are affected by the aging of eggs in particular. Some studies have focused on the aging of cultured oocytes, which could cause increased cytogenetic abnormalities^11–13^, fewer available cleavage embryos and poorer PR. However, these studies have not proposed effective ways to eliminate the effects of egg aging in r-ICSI cycles. Increasing numbers of studies have shown that blastocyst culture is an effective method of obtaining “good development potential” embryos and results in satisfactory clinical outcomes^14–17^. It is possible that blastocyst culture may give the same result for r-ICSI and be an effective strategy to obtain satisfactory results. Embryos from r-ICSI may be cultured to the blastocyst stage and cryopreserved, instead of cleavage embryos being frozen, and subsequently used in cryopreservation-warming cycles. Similarly, blastocyst culture results in a decrease in the number of embryo transfer cycles, which may affect the final cumulative pregnancy rate. To address these issues, the current study involved a retrospective analysis of a total of 625 r-ICSI cycles performed using freeze-all-embryos (from January 2013 to December 2016) and 144 cleavage-stage embryo transfers and 188 blastocyst-stage transfers in subsequent cryopreservation-warming cycles (from March 2013 to December 2018) in a single infertility center.

## Results

For r-ICSI frozen cleavage, 502 available embryos were cryopreserved in the 132 cycles and 345 embryos were used for transfer in 144 subsequent cryopreservation-warming cycles, finally resulting in 24.31% PR, 11.59% IR, 19.44% LBR and 31 newborns (no anomalies). For r-ICSI frozen blast, 2537 embryos were cultured to blastocyst stage and 297 (11.71%) embryos reached a grade of 3BB or better quality and were cryopreserved in 158 cycles. A total of 249 embryos were used for transfer in 188 subsequent cryopreservation-warming cycles, finally resulting in 46.27% PR, 40.56% IR, 37.77% LBR and 78 newborns (one microtia) (Table 1).

**Table 1:** Comparisons of patient conditions and outcomes of ET between r-ICSI frozen cleavage and r-ICSI frozen blast.

	r-ICSI frozen cleavage (N = 144)	r-ICSI frozen blast (N = 188)	P
Maternal age (year) at oocyte retrieval	32.41 ± 4.68	31.38 ± 4.14	0.034
Maternal BMI	21.83 ± 2.85	22.69 ± 3.08	0.010
Gonadotropin dose	2830.56 ± 1276.43	2465.69 ± 1073.86	0.005
Number of oocyte retrieval	14.27 ± 6.31	14.75 ± 6.63	0.501
Number of oocytes injected ( mature oocytes: MII )	11.84 ± 5.43	12.17 ± 5.96	0.607
Number of MI	1.58 ± 0.72	1.69 ± 0.83	0.457
Number of GV	0.85 ± 0.37	0.89 ± 0.39	0.874
PR	24.31 (35/144)	46.27 (87/188)	0.000
IR	11.59 (40/345)	40.56 (101/249)	0.000
MR	20.00 (7/35)	18.39 (16/87)	0.837
LBR	19.44 (28/144)	37.77 (71/188)	0.000
Newborns (N)	31	78^a^	–

Data are % (n/N) or the mean ± SD. PR: pregnancy rate; IR: implantation rate; MR: miscarriage rate; LBR: live birth rate.

^a^One microtia.

Clinical outcomes for the method of r-ICSI frozen blast were better than those for the method of r-ICSI frozen cleavage in calculations for cryopreservation-warming ET cycles (PR 24.31% vs. 46.27%, respectively; P = 0.000; IR, 11.59% vs. 40.56%, respectively; P = 0.000; LBR, 19.44% vs. 37.77%, respectively; P = 0.000). Patient condition was also significantly different between the two strategies (Table 1). Furthermore, the results for LBR remained better using the method of r-ICSI frozen blast after logistic regression analyses to control for confounding factors, when compared with the method of r-ICSI frozen cleavage (OR = 2.721, 95% CI 1.604–4.616, P < 0.001) (Table 2). Patient condition and conservative cLBR were compared between the method of r-ICSI frozen cleavage and method of r-ICSI frozen blast with regard to the number of oocyte retrieval cycles (Table 3) (17.39% vs. 15.30%, respectively; P = 0.576). Similar results were obtained for conservative LBR after logistic regression analyses were performed to adjust for other confounding factors (OR = 0.925, 95% CI 0.557–1.535, P = 0.762) (Table 2).

**Table 2:** Binary logistic regression analysis of factors associated with LBR in ET cycles and conservative cLBR in oocyte retrieval cycles.

	LBR OR (95%CI)	P value	cLBR OR (95%CI)	P value
Maternal age (year) at oocyte retrieval	0.956 (0.900–1.016)	0.148	0.960 (0.909–1.015)	0.148
Maternal BMI	0.973 (0.896–1.057)	0.519	0.982 (0.916–1.054)	0.619
**Ovarian stimulation protocol**				
GnRH agonist long	Reference		Reference	
GnRH agonist short	1.432 (0.579–3.545)	0.437	1.102 (0.476–2.555)	0.820
GnRH antagonist	0.638 (0.358–1.137)	0.127	0.699 (0.413–1.184)	0.182
Gonadotropin dose	1.000 (1.000–1.000)	0.564	1.000 (1.000–1.000)	0.149
Number of oocyte retrieval	0.996 (0.956–1.036)	0.830	1.085(1.045–1.127)	0.000
**Method**				
r-ICSI frozen cleavage	Reference		Reference	
r-ICSI frozen blast	2.721 (1.604–4.616)	0.000	0.925 (0.557–1.535)	0.762

**Table 3:** Comparison of patient conditions and conservative cLBR between r-ICSI frozen cleavage and r-ICSI frozen blast groups.

	r-ICSI frozen cleavage (N = 161)	r-ICSI frozen blast (N = 464)	P
Maternal age (year) at oocyte retrieval	32.91 ± 4.77	32.68 ± 4.69	0.594
Maternal BMI	22.17 ± 3.26	22.67 ± 3.43	0.108
**Ovarian stimulation protocol**			
GnRH antagonist	52	188	0.065
GnRH agonist long	89	239	0.409
GnRH agonist short	19	34	0.079
Microsimulation	1	3	0.972
Gonadotropin dose	3049.92 ± 1322.81	2851.45 ± 1320.73	0.101
Number of oocyte retrieval	12.07 ± 6.18	11.29 ± 5.77	0.147
Conservative cLBR	17.39 (28/161)	15.30 (71/464)	0.576

Data are % (n/N) or the mean ± SD. cLBR: cumulative live birth rate.

## Discussion

TFF is unavoidable during conventional IVF cycles and many studies have recommended that r-ICSI be performed when it happens^1^. Nevertheless, it is known that a low normal fertilization and a poor PR occur following ET in fresh cycles. Therefore, some embryologists do not recommend performing r-ICSI and it was believed that r-ICSI merely increases the fertilization rate and the number of embryos, but is not helpful for clinical treatment. In this study, conservative LBR of r-ICSI could reach 17.39% (method 1) and 15.30% (method 2), so it is valuable to perform r-ICSI when using freeze-all-embryos, and the view people have held for a long time that r-ICSI has no clinical application may need to be changed.

In a previous study^9^, it was found that an acceptable PR can be obtained by using cryopreserved cleavage embryos in subsequent cryopreservation-warming cycles for r-ICSI. The method of using cryopreservation-warming cycles partially solves the problems resulting from asynchrony of the embryo and endometrium. At the same time, we need to consider the fact that the PR of the study was about 25–30%, which is significantly lower than that of the conventional cycle. It may be that only cleavage embryos were used in the study, and that the developmental potential of those embryos was poor due to the aging of the eggs. Normally, the optimal fertilization time for a human is at < 24 h after ovulation in vivo. If the oocyte is not fertilized within the limits of the optimal fertilization time, the unfertilized oocytes begin the process of apoptosis and degradation known as post-ovulatory oocyte aging^18^. In vitro, oocytes of day 1 r-ICSI begin to undergo the process of post-ovulatory oocyte aging because r-ICSI is generally performed about 24 h after oocyte retrieval, although there may be some differences in vivo and in vitro.

Given ethical concerns and the limited availability of material, only a few articles have focused on the negative effects of aging human eggs due to r-ICSI^11–13^, and the results were not confirmed by sufficient evidence. Non-human animal research focusing on oocyte aging may provide more sufficient and direct evidence for the species concerned. It has been reported that in vitro postovulatory aging of oocytes may impair oocyte quality, resulting in lower fertilization and blastocyst formation rates. Aging of oocytes interferes with numerous molecular, biochemical, morphological and organelle alterations. Negative changes in oocytes have been found in the zona pellucida, oolemma, the structure of the plasma membrane, mitochondria, cortical granules, cytoskeleton, and spindle and chromosome alignment^18–20^. Recent studies have shown that postovulatory oocyte aging has a negative influence on localization and expression of maternal effect proteins, the dynamics of mRNA, pericentromeric proteins and spindle integrity^21^ and, in particular, resulted in destabilization of the spindle assembly checkpoint, causing aneuploidy^22^. The final results are lower fertilization and blastocyst formation rates and poor PR. However, PR may be obtained and a baby born, which shows that a proportion of eggs from day 1 r-ICSI may be affected minimally or not at all by postovulatory oocyte aging. How to select the potentially developing embryos derived from these eggs for transfer is a question considered in our clinical work.

As mentioned above, embryos derived from aging oocytes may be more likely to carry abnormal chromosomes, due to aneuploidy caused by postovulatory aging. Some studies have also found that r-ICSI cleavage embryos have a high rate of chromosomal abnormalities^12,13^. It is well-known that embryos with chromosomes abnormalities will more likely fail to form blastocysts and that blastocyst culture is an effective method for reducing the number of embryos with chromosome abnormalities and obtaining more embryos with normal karyotype^23,24^. In this study, the outcomes (PR, IR and LBR) for r-ICSI frozen blast were significantly better than those for r-ICSI frozen cleavage. In addition, the rate of available blastocysts after r-ICSI embryo culture was 11.71%, which is much lower than the available blastocyst formation rate in conventional cycles. These results show that, for r-ICSI cycles, embryos with a poor developmental potential will be eliminated after blastocyst culture and blastocyst transfer can significantly improve the clinical outcomes. In a word, blastocyst culture can select “good” embryos to solve the problem of embryo development potential, and transfer in cryopreservation–warming cycles can solve the asynchrony of embryo and endometrium. Therefore, this is an effective method for r-ICSI; instead of cryopreserving cleavage embryos, embryos are cultured to the blastocyst stage, cryopreserved, and subsequently used in cryopreservation-warming cycles. However, it should be noted that embryonic chromosome abnormalities cannot be completely avoided when selecting embryos from blastocyst cultures only^25^, therefore it is also suggested that r-ICSI should be applied in combination with preimplantation genetic testing.

Of note, the method of frozen blast neither improves, of course, nor decreases the final outcome of treatment based on the results of comparable conservative cLBR between r-ICSI frozen blast and r-ICSI frozen cleavage in this study. However, blastocyst transfer can indeed decrease the number of transfer cycles, improve the efficiency of treatment, and save time and cost of patients’ medical treatment. Therefore, it is recommended that a method of r-ICSI frozen blast is performed in r-ICSI.

In the study, although satisfactory outcomes could be achieved through the method of r-ICSI frozen blast, there were just 34.05% cycles with frozen blastocysts because the rate of blastocyst formation is very low. It is therefore necessary to inform patients that nothing may be achieved using this method, not even a chance of ET, after paying the costs of ICSI and blastocyst culture. In addition, although there is no evidence that blastocyst culture has a negative impact on the offspring at present, it is prudent to note that there have been some differences reported between babies born following cleavage-stage ET and blastocyst ET^26^. Finally, whether blastocyst transfer can fully eliminate the effect of egg aging on offspring requires further study, although the newborns reported in this study were healthy, except for one malformation. All of the above need to be fully considered and the patients informed.

## Conclusions

The results of this study confirmed that it was of value to perform r-ICSI if using freeze-all-embryos because of the 15% and 17% cumulative LBR, and this may change the long-held view that r-ICSI has no clinical application. Furthermore, blastocyst culture is an effective way to select r-ICSI embryos with developmental potential for transfer. If r-ICSI is decided upon, it is recommended that r-ICSI embryos are cultured to the blastocyst stage, cryopreserved, and used in subsequent cryopreservation-warming cycles. This can not only have the same satisfactory results, but also save time and the cost of patients’ medical treatments. Based on this study, the transfer of r-ICSI embryos was considered safe, but further studies involving long-term follow-ups are needed.

## Materials and methods

### Patients and cycles

**Figure 1:**
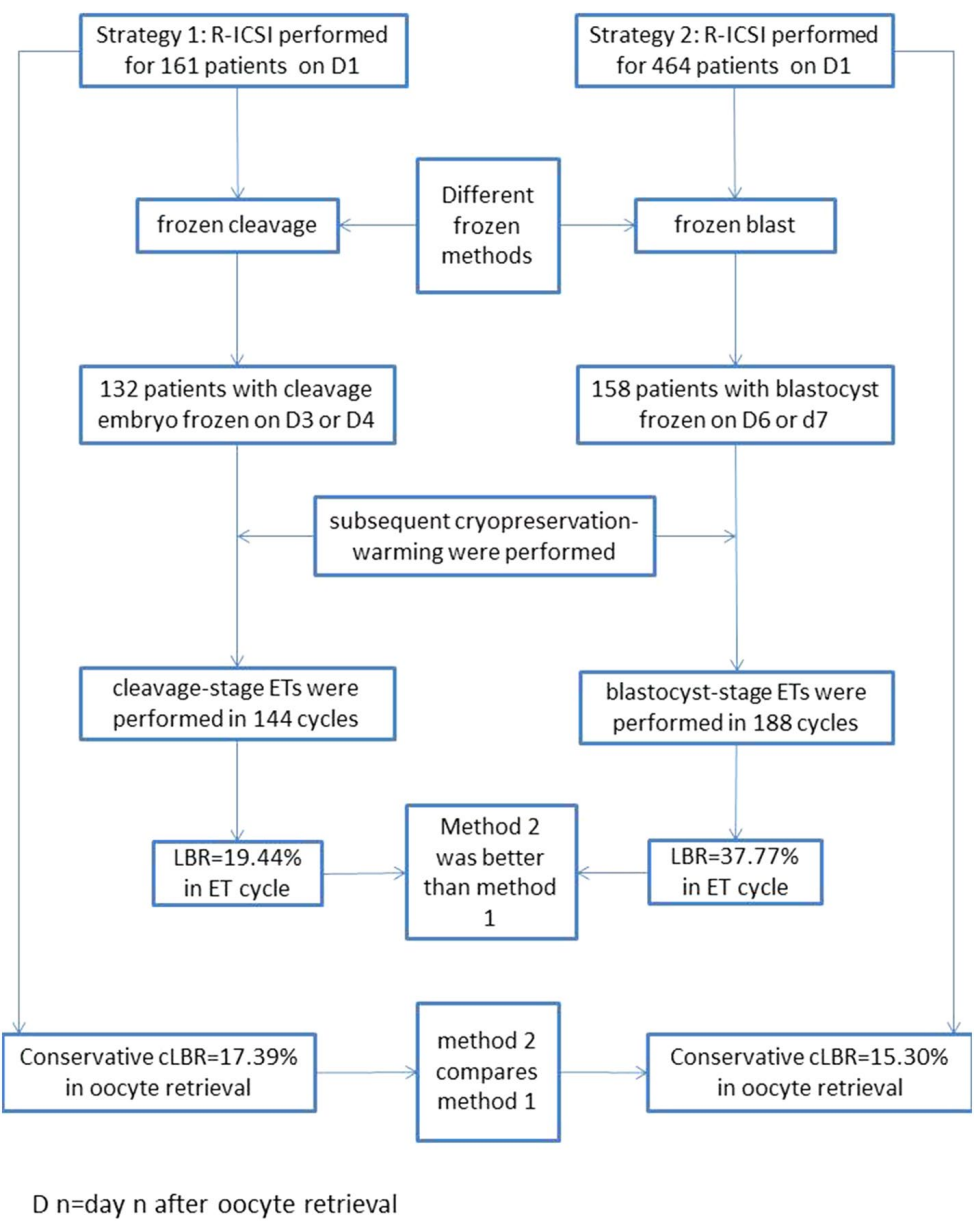
Flow chart and outcomes of method 1 and method 2.

This was a retrospective analysis of the clinical outcomes of embryos derived from r-ICSI cycles, which were performed after TFF in conventional cycles; 27,582 individuals used conventional IVF in this infertility center from January 2013 to December 2016. Prospective randomized controlled studies to determine which method is better for cleavage embryos (frozen or blastocyst frozen) are difficult to perform in r-ICSI cycles because the occurrence of TFF is unpredictable and it is also very upsetting to patients. Therefore, two transfer methods using freeze-all-embryos were included in the study. Method 1: cleavage embryos from r-ICSI were frozen directly, without fresh ETs, on day 3 or 4 after oocyte retrieval; the cleavage embryos were transferred in a subsequent cryopreservation-warming cycle (r-ICSI frozen cleavage). In total, 161 cycles adopted this method and 132 cycles obtained at least one embryo to be frozen. Similarly, method 2, with directly frozen blastocysts (r-ICSI frozen blast), was used for 464 cycles, and 158 cycles obtained at least one blastocyst to be frozen on day 6 or 7 after oocyte retrieval. During subsequent cryopreservation-warming cycles, 144 cleavage-stage ETs occurred with r-ICSI frozen cleavage and 188 blastocyst-stage ETs were performed with r-ICSI frozen blast (Fig. 1).

The study was approved by the Ethics Committee of Beijing University Third Hospital (reference no: 2016SZ-071) in November 26, 2016, all methods were performed in accordance with the relevant guidelines and regulations and all patients signed written informed consent.

### Fresh cycles

All participants used short or long protocols for ovarian stimulation. Between 36 and 38 h after hCG administration, the oocytes were retrieved and collected into a four-well dish which contained fertilization medium (HTF, LifeGlobal, USA, G-IVF, Vitrolife, Sweden or Sydney IVF medium, Cook, Australia) and were cultured in incubators at 37 °C under 5 or 6% CO_2_, and 3–4 h later, inseminated in conventional IVF cycles. Spermatozoa were collected by the swim-up technique, using 50,000 motile spermatozoa per insemination dish. Normal fertilization was identified by the presence of 2PN 16–19 h after insemination. When TFF was detected, r-ICSI was performed as previously described^27^. Spermatozoa from the original insemination were used in rescue ICSI procedures. Oocytes were assessed for evidence of fertilization 20–22 h after r-ICSI. In this study, embryos from r-ICSI were frozen directly, without fresh ETs. In r-ICSI frozen cleavage, based on the number of cells of the embryos and cytoplasmic fragmentation, embryos were frozen either on day 3 after oocyte retrieval (on day 2 after r-ICSI) or on day 4 after oocyte retrieval (on day 3 after r-ICSI). Embryos were frozen on day 3 after oocyte retrieval when there were the only sufficient embryos (≥ four-cells, ≤ 10% fragmentation) to be frozen. In other cases, for example when there were some two-cell or three-cell embryos on day 3 after oocyte retrieval, embryo culture was continued for one additional day. If embryos continued developing for 1 day and available embryos were obtained (≥ 4 cells and ≤ 10% fragmentation), the developing embryos were frozen on that day (day 4 after oocyte retrieval). Likewise, embryos were cultured to the blastocyst stage when there were at least one- or two-cell embryos on day 3 after oocyte retrieval. Blastocysts that reached a grade of 3BB or better quality were cryopreserved by day 6 or 7 after oocyte retrieval in r-ICSI frozen blast. The vitrification protocols were performed by standard procedures for cleavage embryos and blastocysts, as described elsewhere^28,29^ (Fig. 1).

### Vitrification-warming cycles

ETs for cryopreservation-warming cycles were performed in either artificial hormone replacement cycles or natural monitored cycles. Natural cycles were used for women with regular ovulatory menstruation. Thawed ET was scheduled for 2, 3 or 5 days after ovulation. Luteal support was given using intramuscular injections of progesterone (progesterone; Shanghai General Pharmaceutical Company, China) in oil (20–40 mg) from the night of transfer to day 12, 14 or 15, when serum hCG levels were assessed. Endometrial preparation was used, with oral estradiol, in artificial hormone replacement cycles. When endometrial thickness and estradiol concentrations were suitable, this phase was complemented by administration of progesterone. ET was performed on day 7 of progesterone administration. Hormone replacement therapy was continued until the pregnancy test. Serum hCG concentration was measured 12 days after embryo replacement. One week later, transvaginal ultrasound was performed to confirm intrauterine pregnancy. In cases of pregnancy, steroid supplementation was maintained until week 12 of gestation.

### Outcome parameters and statistical analysis

Clinical pregnancy (PR) was defined by the detection of a gestational sac on ultrasound examination at least 5 weeks after transfer. The implantation rate (IR) was calculated as the number of gestational sacs divided by the number of embryos transferred. The miscarriage rate (MR) was calculated as the number of miscarriage cycles divided by the number of transferred cycles. Live birth rate (LBR) was calculated as the number of live delivery cycles divided by the number of transferred cycles. The conservative cumulative LBR (cLBR) assumes that patients who did not return for treatment did not have a pregnancy resulting in a live birth. The conservative cLBR estimate was calculated as the number of live births divided by the number of oocyte retrieval cycles^30^. There were just two patients without a live birth who did not return for treatment in the r-ICSI frozen cleavage group, and three patients in the r-ICSI frozen blast group. Thus, only the conservative cLBR were used in this study. All statistical analyses were performed with SPSS software (SPSS Inc., Chicago, IL, USA). The basic characteristics of the patients were compared using the Student’s *t*-test (continuous variables), while categorical variables were evaluated by chi-squared tests. Statistical significance was defined as a P-value < 0.05. Binary logistic regression analyses were used to evaluate the possible relationship between LBR (or CLBR) and different strategies performed, after adjusting for other potential confounding factors, including parental age, parental BMI, ovarian stimulation protocol, gonadotropin dose, and number of oocytes retrieved.

## Data Availability

All data generated or analyzed during this study are available from the corresponding author on reasonable request.
